# BCDIN3D regulates tRNA^His^ 3’ fragment processing

**DOI:** 10.1371/journal.pgen.1008273

**Published:** 2019-07-22

**Authors:** Calder W. Reinsborough, Hélène Ipas, Nathan S. Abell, Ryan M. Nottingham, Jun Yao, Sravan K. Devanathan, Samantha B. Shelton, Alan M. Lambowitz, Blerta Xhemalçe

**Affiliations:** 1 Department of Molecular Biosciences, Institute for Cellular and Molecular Biology, University of Texas at Austin, Austin TX, United States of America; 2 Department of Genetics, Stanford University School of Medicine, Stanford, CA, United States of America; Ohio State University, UNITED STATES

## Abstract

5’ ends are important for determining the fate of RNA molecules. BCDIN3D is an RNA phospho-methyltransferase that methylates the 5’ monophosphate of specific RNAs. In order to gain new insights into the molecular function of BCDIN3D, we performed an unbiased analysis of its interacting RNAs by Thermostable Group II Intron Reverse Transcriptase coupled to next generation sequencing (TGIRT-seq). Our analyses showed that BCDIN3D interacts with full-length phospho-methylated tRNA^His^ and miR-4454. Interestingly, we found that miR-4454 is not synthesized from its annotated genomic locus, which is a primer-binding site for an endogenous retrovirus, but rather by Dicer cleavage of mature tRNA^His^. Sequence analysis revealed that miR-4454 is identical to the 3’ end of tRNA^His^. Moreover, we were able to generate this ‘miRNA’ *in vitro* through incubation of mature tRNA^His^ with Dicer. As found previously for several pre-miRNAs, a 5’P-tRNA^His^ appears to be a better substrate for Dicer cleavage than a phospho-methylated tRNA^His^. Moreover, tRNA^His^ 3’-fragment/‘miR-4454’ levels increase in cells depleted for BCDIN3D. Altogether, our results show that in addition to microRNAs, BCDIN3D regulates tRNA^His^ 3’-fragment processing without negatively affecting tRNA^His^’s canonical function of aminoacylation.

## Author summary

We previously identified an important modification of human microRNAs, written by BCDIN3D, an RNA phospho-methyltransferase linked to triple negative breast cancer, obesity and type II diabetes. Here, we employed a powerful sequencing method that overcomes RNA secondary structure and RNA modifications, to unbiasedly identify RNAs stably bound to BCDIN3D. This analysis showed that BCDIN3D interacts with full-length phospho-methylated tRNA^His^ and with miR-4454. Close inspection of miR-4454 sequence revealed that it is identical to the tRNA^His^ 3’ end, and we present evidence that miR-4454 is in fact a tRNA fragment generated through Dicer cleavage. Overall, our results indicate that tRNA^His^ interaction with BCDIN3D plays non-canonical roles, one of which is to regulate the generation of tRNA^His^ 3’ fragments. This may have important biological implications as ‘miR-4454’ has been identified as a potential biomarker in several human diseases. Our work also provides a compelling example of how RNA modifiers and RNA processing enzymes multi-task using different species of small RNAs, such as miRNAs and tRNAs.

## Introduction

The 5’ ends of eukaryotic RNAs are vital for determining their processing and function [1]. The most well-known 5’ end RNA modification is the m^7^G cap, which has multiple functions, including of protecting messenger RNAs from exonucleolytic degradation, and of marking them for nucleo-cytoplasmic export and translation [2]. Additionally, a chemically simpler 5’ end modification by O-methylation occurs directly on 5’ phosphate ends, either on the γ-phosphate of nascent tri-phosphorylated snRNAs, or on the α-phosphate of processed monophosphate RNAs [3]. These methylations are carried out by the Bicoid Interacting 3 (BIN3) [4] family of methyltransferases, which are conserved from fission yeast to humans [5]. In humans, two BIN3 related enzymes, MePCE and BCDIN3D, have been identified. Jeronimo *et al*., uncovered that MePCE methylates the γ-phosphate of the 7SK snRNA [6], while we discovered that BCDIN3D methylates the 5’ monophosphate of two specific microRNA precursors, pre-miR-145 and pre-miR-23b, both *in vitro* and in cells, to inhibit their processing into mature miRNA by Dicer [5]. Our initial analysis in MCF-7 cells also suggested that other microRNAs could be methylated by BCDIN3D [5]. However, due to lack of methods to specifically enrich 5’ phospho-methylated RNAs, the methylation of other types of RNAs with 5’ monophosphates was not analyzed in our initial study.

MePCE forms a stable ribonucleoprotein complex with its 7SK small nuclear RNA target [6]. Based on these findings, we hypothesized that the other member of the BIN3 family, BCDIN3D, may interact with at least a subset of its RNA targets in a similar manner. Purification and sequencing of RNAs interacting with BCDIN3D revealed that BCDIN3D specifically interacts with mature tRNA^His^ and miR-4454, which our results suggest to be a tRNA^His^ 3’ fragment derived from cleavage of mature tRNA^His^. Importantly, BCDIN3D downregulation results in increased levels of tRNA^His^ 3’ fragments in cells. Overall, our results indicate that tRNA^His^ interaction with BCDIN3D plays non-canonical roles, one of which is to regulate the generation of tRNA^His^ 3’ fragments. This may have important biological implications as miR-4454 has been identified as a potential biomarker in several human diseases [7–9]. Moreover, given that tRNA 3’ fragments limit the mobility of transposable elements in mammalian cells [10], BCDIN3D may impact genomic stability.

## Results

### BCDIN3D stably interacts with small RNAs

**Fig 1:**
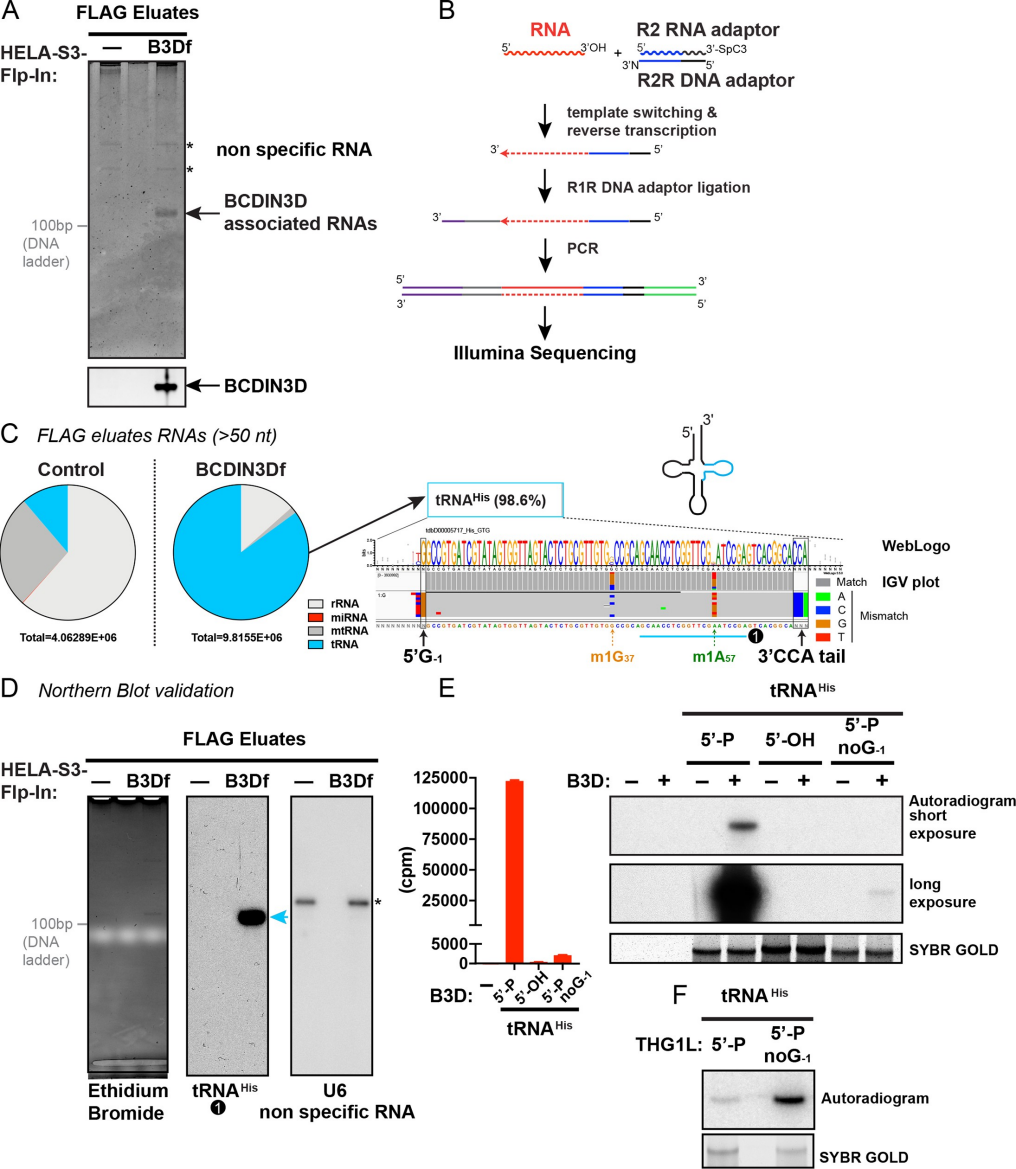
BCDIN3D stably interacts with mature tRNA^His^. A. RNAs purified from HeLa-S3-Flp-In and HeLa-S3-Flp-In-BCDIN3Df FLAG eluates were analyzed on a denaturing 15% polyacrylamide/urea gel stained with silver. The asterisks indicate non-specific RNAs. A 10 bp DNA Ladder was run in an adjacent lane and the position of its 100 bp band is indicated. Please note that the migration of the 10 bp ladder is offset by 30 to 50 nt compared to RNA. B. Schematic of TGIRT-seq protocol used to generate the next generation sequencing libraries. C. Results of the TGIRT-seq libraries from BCDIN3Df-interacting small RNAs (reads > 50nt) from two biological repeats. The bio-informatic pipeline is schematized in S1 Fig, and full results of the two biological repeats are shown in S1 Table. The sequences of BCDIN3D interacting RNAs >50nt mapping to tdbD00005717_His_GTG (tRNA^His^) from one of the two biological repeats are represented in a WebLogo format (top) and in an IGV plot representation (bottom) that includes coverage plots to the reference sequence. The numbers of reads are indicated to the left of the coverage plot in brackets. Nucleotides in reads matching nucleotides in the reference are colored in gray, while mismatched nucleotides are color coded by nucleotide (A, green; C, blue; G, brown; and T, red). The nontemplated T residues at the 5’ end of tRNA^His^ correspond to nontemplated A nucleotide addition by TGIRT at cDNAs 3′ ends. Highlighted are the 5’ G_-1_ and the 3’ CCA tail characteristic of mature tRNA^His^, as well as the sites of m^1^G_37_ and m^1^A_57_ modifications that lead to the distinctive nucleotide misincorporation by TGIRT. D. Validation of the TGIRT-seq results with northern blotting using tRNA^His^ northern blot probe #1, the target position of which is shown in cyan in Fig 1C. The Northern blot with the U6 probe is to show equal background RNA in Control and BCDIN3Df FLAG eluates. E. BCDIN3D prefers to methylate mature tRNA^His^-5’P containing the 5’ G_-1_ residue. The indicated synthetic tRNA^His^ RNAs were *in vitro* methylated with BCDIN3D using radioactive [^3^H]-SAM as methyl group donor. The bottom panel shows the SYBR Gold stained gel that was used for the autoradiography in the top panels, while the graph shows the scintillation counts of C[^3^H]_3_ incorporated into the RNA. F. THG1L prefers to guanylate tRNA^His^ without the 5’ G_-1_ residue. tRNA^His^-5’P with or without the G_-1_ residue were *in vitro* guanylated with recombinant THG1L using radioactive [^32^P]α-GTP. The bottom panel shows the SYBR Gold stained gel that was used for the autoradiography in the top panel.

In order to purify RNAs interacting with BCDIN3D, we used HeLa-S3-FlpIn cells containing a single insertion at a FRT locus of BCDIN3D tagged with a FLAG tag at its C-terminus (denoted BCDIN3Df or B3Df). We first purified BCDIN3Df with a protocol that combines FLAG antibody co-immunoprecipitation, followed by FLAG peptide elution. We previously used this same protocol to show an RNase A-sensitive interaction of BCDIN3Df with Dicer [5]. We then extracted RNAs from FLAG eluates of Control and BCDIN3Df cells with a protocol that allows recovery of RNAs of all sizes (see Methods). Analysis of the extracted RNAs on a denaturing 15% polyacrylamide/urea gel stained with silver showed a prominent band of 70 to 90 nucleotides in the FLAG eluates from BCDIN3Df cells, along with lighter non-specific bands present in the FLAG eluates from both Control and BCDIN3Df cells (Fig 1A).

### TGIRT-seq identifies mature tRNA^His^ as the major small RNA stably interacting with BCDIN3D

In order to unbiasedly uncover the identity of the small RNAs interacting with BCDIN3D, we sought to use next generation sequencing (NGS). Commonly used small RNA-Seq protocols use RNA ligases to add RNA adaptors to each end of the RNA prior to reverse transcription into cDNA and NGS library amplification. However, RNAs methylated by BCDIN3D cannot be amplified by these protocols because the dimethyl-phosphate (5’Pme2) is resistant to ligation by RNA ligases [5]. In an attempt to circumvent the 5’Pme2 end ligation problem, we first used a small RNA-Seq protocol that employs T4 Rnl2tr (T4 RNA ligase 2, truncated) to add an adaptor only on the 3’ end, followed by reverse transcription and cDNA circularization with CircLigase prior to amplification [11], but our attempts were unsuccessful. We then reasoned that RNA secondary structure could also prevent efficient amplification of BCDIN3D-interacting RNAs. Therefore, we used a TGIRT-seq protocol that combines template switching to the RNA 3’ end and a highly processive Thermostable Group II Intron Reverse Transcriptase [12] for cDNA synthesis (Fig 1B). The use of this method resulted in successful library amplification.

Because the visible BCDIN3D-specific RNA band is 70 to 90 nt, we first focused on paired end reads longer than 50 nt (Fig 1C and S1 Table). The reads were dominated by mature tRNA^His^, containing both the non-templated 5’ G_-1_ characteristic of tRNA^His^, and the non-templated 3’ CCA tail [13] (see WebLogo and IGV plot of obtained sequences in Fig 1C). In addition to tRNA^His^, the BCDIN3D-interacting RNA libraries also included other reads corresponding to RNAs >50 nt, but these were found at the same level in the Control libraries (Fig 1C and S1 Table), and were consequently deemed background reads. We confirmed our TGIRT-seq results by northern blotting with a specific tRNA^His^ probe (probe #1) that is complementary to the TΨC arm (Fig 1C and 1D and S4 Table). Notably, this probe detected full-length tRNA^His^ molecules present exclusively in the BCDIN3Df FLAG eluates, while a U6 probe detected a background U6 RNA in both samples (Fig 1D).

As mentioned above, our TGIRT-seq results show that BCDIN3D exclusively interacts with mature tRNA^His^ containing the non-templated 5’ G_-1_ (Fig 1C). Consistent with this observation, our *in vitro* RNA methyltransferase assays with recombinant BCDIN3D show that BCDIN3D has a marked preference for mature tRNA^His^ containing the 5’ G_-1_, when compared to a pre-tRNA^His^ without 5’ G_-1_ (Fig 1E), which as expected, is the preferred substrate of tRNA^His^ Guanylyltransferase 1 Like (THG1L) (Fig 1F), the human homolog of yeast Thg1 that adds the non-templated 5’ G_-1_ on pre-tRNA^His^ [14].

### tRNA^His^ interacting with BCDIN3D is phospho-methylated

**Fig 2:**
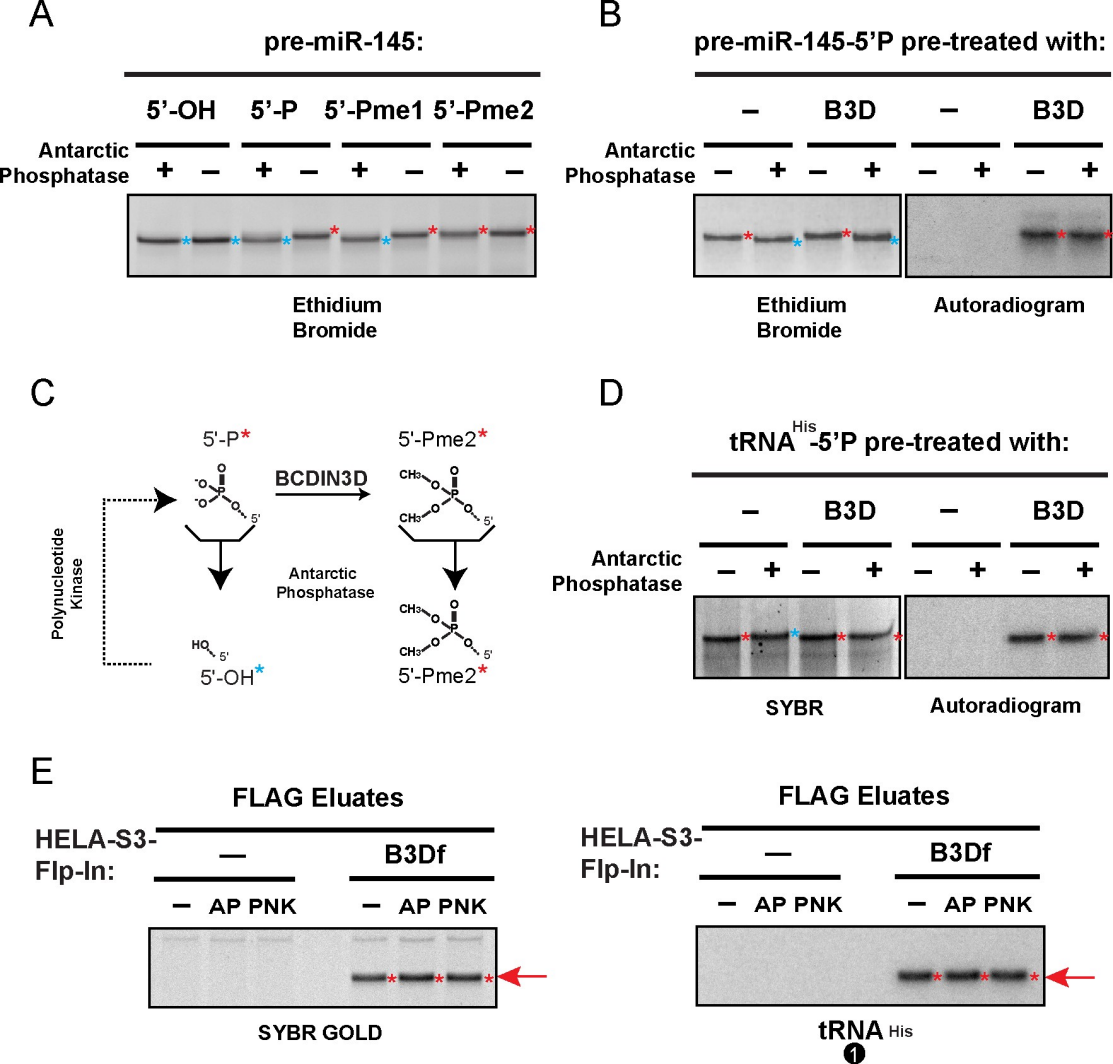
tRNA^His^ interacting with BCDIN3D is phospho-methylated. A. Synthetic pre-miR-145 with either [5’-OH], [5’-P], [5’-Pme1] or [5’-Pme2] ends was treated with Antarctic Phosphatase. Please note that pre-miR-145-5’-OH (cyan *) migrates faster than pre-miR-145 with [5’-P], [5’-Pme1] or [5’-Pme2] ends (red *) on a denaturing 15% polyacrylamide/urea gel. B. Synthetic pre-miR-145 [5’-P] was *in vitro* methylated with BCDIN3D using radioactive [^3^H]-SAM as methyl group donor prior to treatment with Antarctic Phosphatase. The left panel shows the ethidium bromide stained gel that was used for the autoradiography in the right panel, which shows only the *in vitro* methylated RNAs. Please note that only a small fraction of 5’P pre-miR-145 is dimethylated by BCDIN3D in vitro, explaining why AP treatment induces a gel shift in the left panel, which shows the total pre-miR-145, but not in right panel, which shows only the methylated pre-miR-145. C. Schematic showing the 5’ ends and the effect of Antarctic Phosphatase (AP) and Polynucleotide Kinase (PNK). D. Synthetic tRNA^His^-5’-P was *in vitro* methylated with BCDIN3D using radioactive [^3^H]-SAM as methyl group donor prior to treatment with Antarctic Phosphatase. The left panel shows the ethidium bromide stained gel that was used for the autoradiography in the right panel. Please note that tRNA^His^-5’-OH (cyan *) migrates slower than tRNA^His^ with [5’-P], [5’-Pme1] or [5’-Pme2] (red *) ends. E. RNAs purified from HeLa-S3-Flp-In and HeLa-S3-Flp-In-BCDIN3Df FLAG eluates were treated with mock, AP or PNK, and separated on a denaturing 15% polyacrylamide/urea gel and probed with the tRNA^His^ northern blot probe #1. Neither the treatment with AP nor T4 PNK shifted the migration of tRNA^His^ (Fig 1C). The treatment with PNK is a control to verify that the 5’ end is not 5’OH in BCDIN3Df-interacting tRNA^His^.

We next sought to determine whether the BCDIN3Df-interacting tRNA^His^ molecules are methylated on their 5’ ends. Treatment of a synthetic pre-miRNA harboring different 5’ end modifications with an alkaline phosphatase originating from the Antarctic strain TAB5, also called Antarctic Phosphatase [15], can remove 5’P and 5’Pme1 but not 5’Pme2 from the 5’ ends of RNAs, as judged by a distinct electrophoretic mobility shift on the denaturing 15% polyacrylamide/urea gel shown on Fig 2A. When we performed *in vitro* RNA methyltransferase assays with pre-miR-145-5’P using the radioactively labeled methyl group donor S-Adenosyl-Methionine [^3^H]-S-SAM [5], the small fraction of C[^3^H]_3_-methylated pre-miRNA-145 detected by autoradiogram neither disappeared, nor changed its mobility upon treatment with Antarctic Phosphatase (Fig 2B), consistent with our previous results that BCDIN3D dimethylates pre-miRNAs [5]. While this manuscript was in preparation, tRNA^His^ was reported to be monomethylated by BCDIN3D in 293T cells and *in vitro*, based on analysis of RNase A-digested fragments detected by mass spectrometry [16]. Our *in vitro* RNA methyltransferase assay differed from the assay used in [16] in that ours omitted MgCl_2_ and included 5 mM DTT as a reducing agent [5]. As also reported by Blazer *et al*., our conditions ensure optimal BCDIN3D activity *in vitro* [17]. Under our conditions, tRNA^His^ methylated by BCDIN3D *in vitro* becomes fully resistant to Antarctic Phosphatase (Fig 2D). Thus, our data suggest that tRNA^His^ methylated by BCDIN3D is not monomethylated, and is most likely phospho-dimethylated. Furthermore, the migration of tRNA^His^ that co-purified with BCDIN3Df in the FLAG eluates likewise did not change upon treatment with Antarctic Phosphatase, suggesting that tRNA^His^ associated with BCDIN3Df is largely dimethylated in HeLa-S3 cells (Fig 2E).

### BCDIN3D interacts with a miRNA that may be a tRNA^His^ 3’ fragment

**Fig 3:**
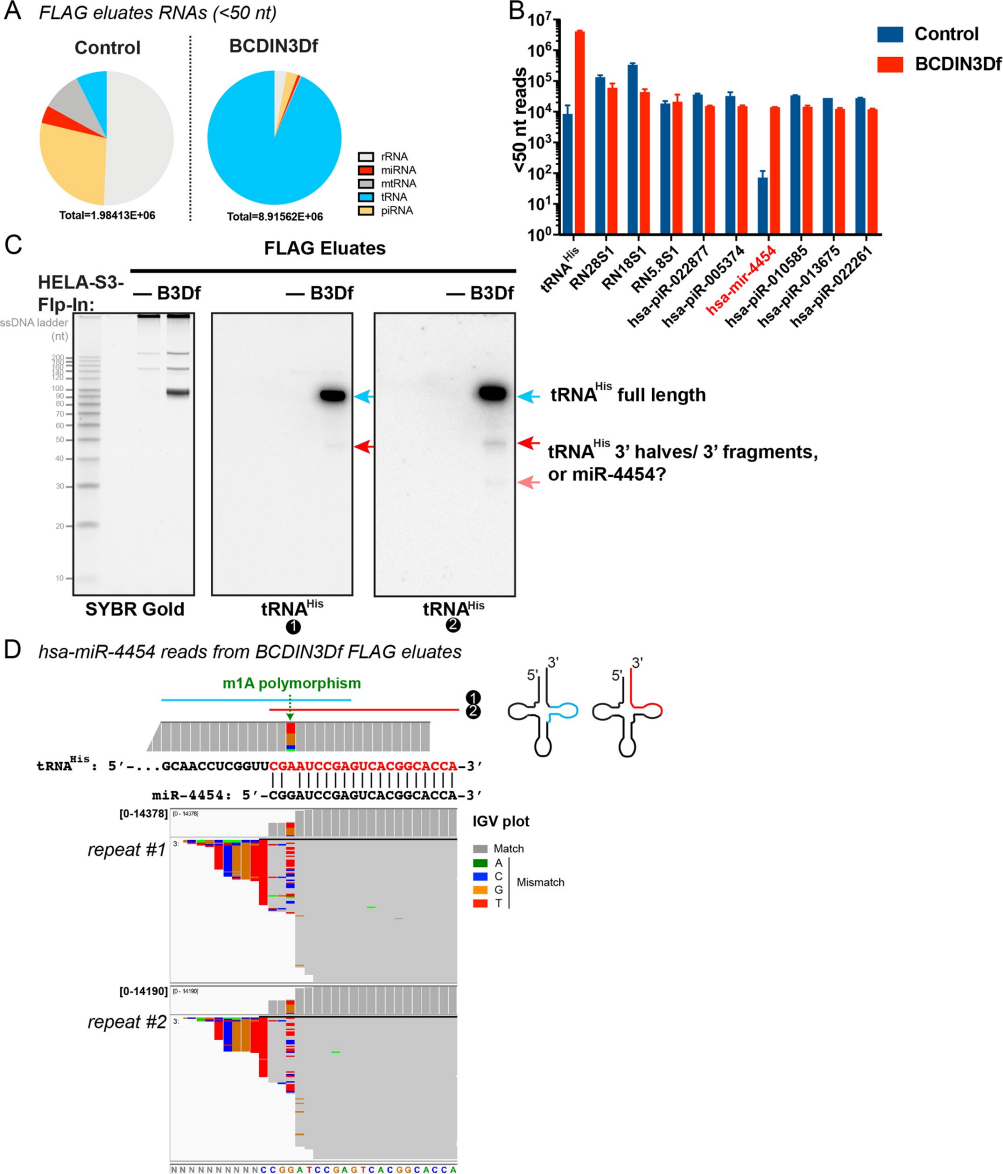
BCDIN3D interacts with a miRNA that may be a tRNA^His^ 3’ fragment. A. Results of the TGIRT-seq libraries from BCDIN3Df-interacting small RNAs (< 50nt) from the same two biological repeats as in Fig 1C. Full results are shown in S3 Table. B. Number of reads < 50nt mapping to the top most abundant RNAs in BCDIN3Df FLAG eluates. Shown are means ±SD of raw reads from two biological replicates. tRNA^His^ (tdbD00005717_His_GTG) and hsa-miR-4454 are the only two top 10 RNAs specifically enriched in BCDIN3Df versus Control FLAG eluates. C. Northern blot analysis of Control and BCDIN3Df FLAG eluates RNAs with tRNA^His^ probes #1 and #2. Note that the ladder is the ss20 ssDNA Ladder and its migration is offset by 10–20 nt compared to RNA. D. Shown are hsa-miR-4454 reads from BCDIN3Df FLAG eluates from two biological repeats in IGV plot representation. Reads are down-sampled to 100 per 50 bp window. Coverage plots are above read alignments to the hsa-miR-4454 reference sequence. The numbers of reads are indicated to the left of the coverage plot in brackets. Nucleotides in reads matching nucleotides in the reference are colored in gray, while mismatched nucleotides are color coded by nucleotide (A, green; C, blue; G, brown; and T, red). Coverage plot of the 3’ end of tRNA^His^ is also shown. The G4 position of the hsa-miR-4454 stem loop aligns to the A57 residue of tRNA^His^ and shows the same nucleotide polymorphism, dominated by T and G that is characteristic of TGIRT-III mis-incorporation at m1A. Please note that A57 in tRNA^His^ corresponds to A58 in other tRNAs due to a skipped position in the variable loop in tRNA^His^.

Our TGIRT-seq analysis revealed a significant number of reads with a length < 50 nt and specific to the BCDIN3Df FLAG eluates (Fig 3A and S3 Table). Some of these reads could be due to a reverse transcription roadblock at modified bases of tRNA^His^ (S2 Fig). For example, while a very large number of reads <50 nt stop just before the G37 residue, no faster migrating band of the expected signal intensity was detected by northern blot with a tRNA^His^ probe #1 (Fig 1D). Although not detected by mass spectrometry [16], tRNA^His^ interacting with BCDIN3D appears to be partially methylated at G37, as evidenced by the observed nucleotide misincorporation introduced by TGIRT at the G37 position (Fig 1C). This is also consistent with the fact that BCDIN3D interacts with TRMT5, which is the enzyme responsible for m^1^G methylation at that tRNA position [18] (S2 Table, S2C Fig).

As shown in Fig 3A and 3B, a significant number of < 50nt reads specific to BCDIN3Df FLAG eluates map to hsa-miR-4454. We validated this result with Reverse Transcription coupled to quantitative PCR (RTqPCR) with a custom Taqman probe for hsa-miR-4454 (S3 Fig). Moreover, a probe complementary to the sequence of this miRNA showed fast migrating bands in northern blots (Fig 3C, tRNA^His^ probe #2), but also a strong signal corresponding to the full-length tRNA^His^. The latter is simply due to the fact that except for a G residue at the position of the m1A modification in the tRNA, the annotated sequence of hsa-miR-4454 is identical to that of the 3’ end of mature tRNA^His^ that includes the 3’ CCA sequence (Fig 3D).

Given the observed interaction between BCDIN3D and tRNA^His^, we hypothesized that hsa-miR-4454 may be a tRNA^His^ 3’ fragment. This hypothesis is particularly appealing for three major reasons: 1) the annotated hsa-miR-4454 does not have an optimal miRNA stem-loop as defined by [19] (S4 Fig); 2) the annotated hsa-miR-4454 genomic locus does not display any feature of transcriptionally active chromatin in HeLa-S3-FlpIn cells (S4B Fig); and 3) the annotated hsa-miR-4454 sequence is the reverse complement of the primer binding site of a HERVH-int LTR-retrotransposon (chr4:163093605–163096486 in hg38 genome assembly), which as its name indicates uses a tRNA^His^ for reverse transcription (S4C Fig).

Close analysis of the hsa-miR-4454 reads from the BCDIN3Df FLAG eluates strongly supports our hypothesis that they originate from tRNA^His^ rather than the annotated genomic locus. Indeed, some hsa-miR-4454 reads from the BCDIN3Df FLAG eluates display heterogenous 5’ ends that differ from the sequence at the annotated MIR4454 genomic locus, but match the sequence of tRNA^His^ (Fig 3D, IGV plots of reads mapping to hsa-miR-4454 from two biological repeats). About 48% of miR-4454 reads are ~18 nt long, and perfectly match tRNA^His^ with a CCA tail (Fig 3D). The rest of the reads are longer and show a high level of polymorphism at what would be the “G4” residue of the annotated hsa-miR-4454 stem loop (Fig 3D and S4D Fig). This residue aligns to the A57 residue of tRNA^His^, which is m1A methylated [16, 20], leading to high levels of polymorphism introduced by TGIRT at that position of tRNA^His^ (Fig 1C) [21, 22]. The spectrum of incorporated nucleotides, dominated by T and G, is characteristic of TGIRT-III mis-incorporation at m^1^A [23] and is similar in both miR-4454 and full-length tRNA^His^ reads (Fig 3D and S4D Fig). Moreover, as mentioned above, a smaller fraction of reads go beyond the annotated hsa-miR-4454 stem loop, and the mismatched nucleotides perfectly align to tRNA^His^ (Fig 3D). Altogether, these observations indicate that the hsa-miR-4454 reads from BCDIN3Df FLAG eluates derive from tRNA^His^.

### Dicer cleavage of tRNA^His^ generates 3’ tRNA fragments corresponding to miR-4454

**Fig 4:**
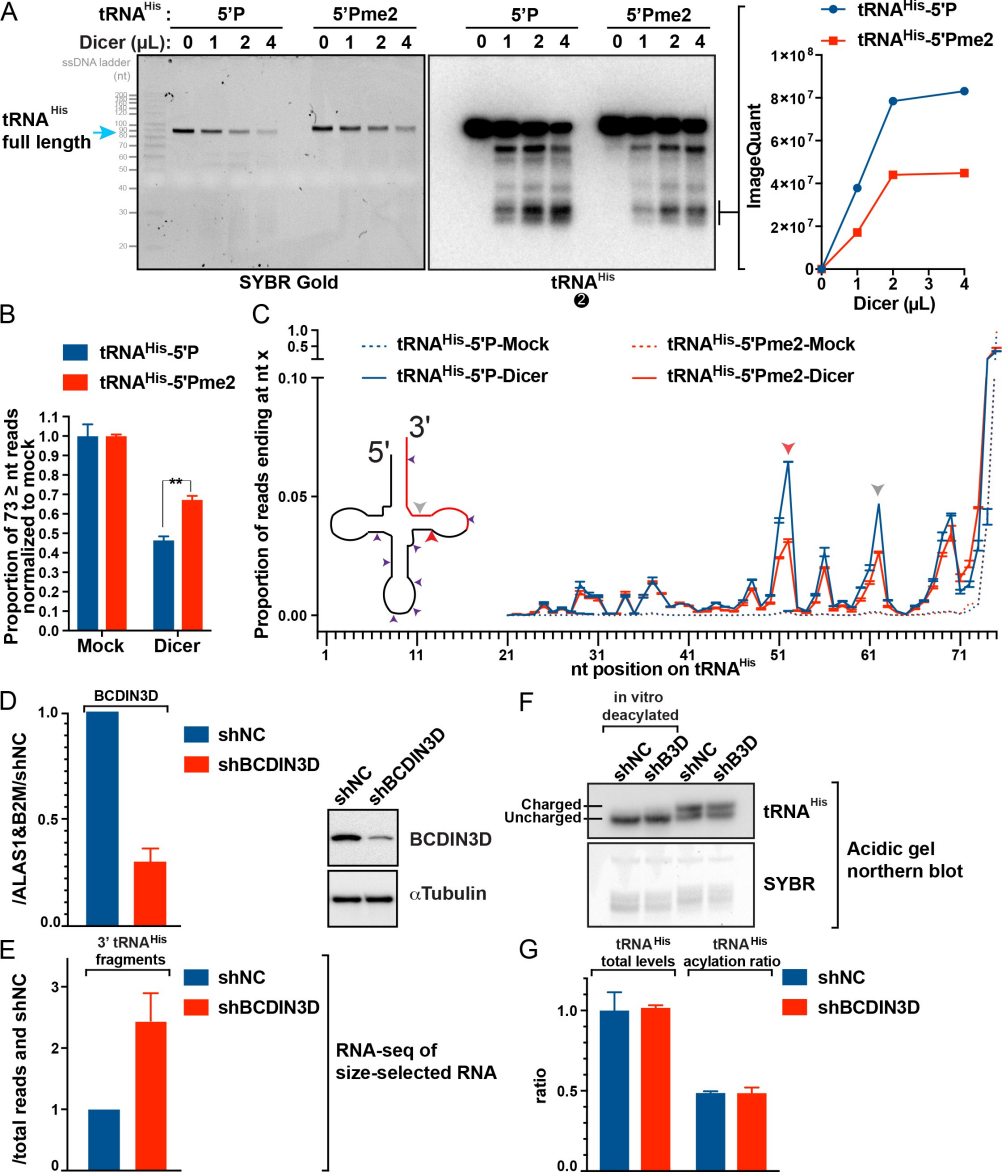
BCDIN3D regulates tRNA^His^ 3’ fragment processing. A. Dicer assay with 5’P and 5’Pme2 tRNA^His^ using 0, 1, 2 and 4 μL of human Dicer at 1 μg/μL. Note that the ladder is the ss20 ssDNA Ladder and its migration is offset by 10–20 nt compared to RNA. The graph on the right shows the quantification by ImageQuant of the indicated bands. B. RNAs from two independent *in vitro* Dicer assays using mock or 2 μl of human Dicer at 1 μg/μl and 20 pmol of tRNA^His^-5’P or -5’Pme2 were sequenced by TGIRT-seq. The bars represent mean ± SD (n = 2) of the ratio of normalized full length tRNA^His^ reads (>73 nt) over the mock control (**, p-value of ~0.0095). C. The graph represents mean ± SD (n = 2) of the ratio of normalized tRNA^His^ reads ending at the indicated position. The deduced Dicer cuts are indicated on the diagram of tRNA^His^ and the sequence of tRNA^His^ aligned to hsa-miR-4454. The red and grey arrows indicate the position of Dicer cuts that are sensitive to tRNA^His^ phospho-methylation. D. The graph on the left shows the RTqPCR analysis of the BCDIN3D mRNA normalized to ALAS1 and B2M, and the images on the right show the western blot analysis of the BCDIN3D protein from MDA-MB-231shNC and shBCDIN3D (shB3D) cells. E. Average ± SEM (n = 2) of tRNA^His^ 3’ fragment reads normalized to total mapped reads relative to shNC. F. Analysis of tRNA^His^ aminoacylation in MDA-MB-231shNC and shBCDIN3D cells. 1 μg of RNAs purified under acidic conditions, which preserve the aminoacyl-tRNA ester bond, was analyzed by acidic gel and northern blot. As a control, 1 μg of RNA was deacylated *in vitro* prior to gel migration. The slower migrating band corresponds to the aminoacylated tRNA^His^ (charged), while the faster migrating band corresponds to the un-aminoacylated tRNA^His^ (uncharged). G. Quantification of acidic northern blot with tRNA^His^ probe from 2 biological replicates, including the acidic northern blot in Fig 4F. The graph shows average ± SEM (n = 2). The total levels of tRNA^His^ are normalized to shNC, and the aminoacylation ratios of tRNA^His^ are calculated as the ratio of the signal of the aminoacylated band over the sum of both bands.

Next, we sought to assess whether hsa-miR-4454 could be generated by Dicer cleavage of tRNA^His^ as previously reported for CCA ending tRNA fragments [24]. In order to test our hypothesis, we performed *in vitro* Dicer processing assays with synthetic 5’P and 5’Pme2 tRNA^His^ in the presence of 1 mM MgCl_2_, as previously described [5] (Fig 4A). These experiments produced several noteworthy observations. The first observation was that incubation with Dicer reduces the amount of visible full-length 5’P-tRNA^His^ (Fig 4A, SYBR Gold stained gel). Interestingly, in contrast to what is observed with Dicer processing of pre-miR-145 into a miRNA duplex [5], no clear RNAs of smaller size were visible in our denaturing polyacrylamide/urea gels stained with SYBR Gold (Fig 4A, left panel) in Dicer- vs mock-treated lanes, suggesting that tRNA^His^ fragments generated by Dicer may be more heterogenous than the miRNA duplex products. Indeed, northern blot with tRNA^His^ northern blot probe #2 showed that Dicer produces a series of discrete 3’ fragments (Fig 4A, tRNA^His^ northern blot) of similar size to the tRNA^His^ 3’ fragments interacting with BCDIN3Df (Fig 3C). Quantification of the faster migrating band by ImageQuant clearly showed an inhibitory effect of 5’Pme2 on the processing of tRNA^His^ 3’ fragments by Dicer (Fig 4A, graph at the right).

In order to examine in detail these effects, we performed TGIRT-seq of RNAs purified from two independent *in vitro* Dicer assays using synthetic tRNA^His^-5’P and -5’Pme2 and 2 μl of Dicer at 1 μg/μl. This setup corresponds to conditions where Dicer reduces the amount of visible full-length 5’P-tRNA^His^ by ~50%. We first quantified the proportion of full-length tRNA^His^ reads present in Dicer-treated vs mock-treated samples (Fig 4B). As expected from the northern blot results, this analysis showed that Dicer treatment reduces the levels of full-length tRNA^His^-5’P by approximately 54%. Most importantly, this same analysis showed that tRNA^His^-5Pme2 is significantly more resistant to Dicer cleavage than tRNA^His^-5’P (**, p-value of ~0.0095). This parallels our previous finding that 5’Pme2 inhibits the processing of pre-miR-145 by Dicer [5].

We then analyzed the TGIRT-seq data to quantify the effect of 5’Pme2 modification on the generation of Dicer cleavage products. To avoid noise from reverse transcription stops and/or incomplete 3’->5’ chemical synthesis of tRNA^His^, we analyzed at which nucleotide position in tRNA^His^ the reads end in mock- and Dicer-treated samples (Fig 4C). As expected, above 90% of reads in the mock-treated samples have 3’ ends corresponding to the last nucleotide of tRNA^His^ (dotted lines in Fig 4C), and this proportion decreases significantly in the Dicer treated samples (solid lines in Fig 4C). Concomitantly, new read ends corresponding to Dicer cuts appear (Fig 4C). These Dicer-specific read ends form several discrete peaks that are shown on the tRNA^His^ sequence and diagram (Fig 4C). Interestingly, two of the major cut sites were significantly decreased in the Dicer-treated tRNA^His^-5Pme2 compared to tRNA^His^-5’P. These two sites are located on the double stranded part of the TΨC arm of tRNA^His^. However, 5’Pme2 only marginally inhibited the generation of a minor cut site in the middle of the loop of the TΨC arm. Thus, 5’ phospho-methylation of tRNA^His^ significantly affects some Dicer cuts, but not others (Fig 4C). As seen in Fig 3D, the reads of tRNA^His^ 3’ fragments interacting with BCDIN3D are also heterogenous and very similar in size to the tRNA^His^ 3’ fragments generated *in vitro* by Dicer. Overall our results suggest that Dicer can generate tRNA^His^ 3’ fragments in a way that is sensitive to the methylation status of the 5’P.

### BCDIN3D knock-down affects the levels of 3’ tRNA^His^ fragments

In order to determine how BCDIN3D knockdown affects the levels of miR-4454/tRNA^His^ 3’ fragments, we analyzed microRNA-Seq data from MDA-MB-231shNC and shBCDIN3D cells. The MDA-MB-231shBCDIN3D cells reduce BCDIN3D mRNA and protein levels by approximately 70% compared to shNC cells (Fig 4D). The dataset was produced from small RNAs < 34 nt extracted from a denaturing 15% polyacrylamide/urea gel (see Methods). When analyzed with a microRNA-Seq bioinformatic pipeline, very few reads mapped to hsa-miR-4454. However, most microRNA-Seq bioinformatic pipelines pre-filter reads mapping to rRNAs and tRNAs. Therefore, we re-analyzed our data with our TGIRT-seq pipeline that does not pre-filter any small RNA reads (S1 Fig). Our analysis revealed a significant number of miR-4454/tRNA^His^ 3’ fragment reads in MDA-MB-231 cells, going up to 473 reads per million in the shBCDIN3D condition, which is comparable to miRNAs well-expressed in MDA-MB-231 cells, such as miR-23a. Importantly, this analysis showed that BCDIN3D depletion significantly increased the levels of tRNA^His^ 3’ fragments (Fig 4E), without affecting either the tRNA^His^ levels, or the steady-state level of tRNA^His^ aminoacylation detected by acidic gel and northern blot (Fig 4F, quantified in Fig 4G) in these cells. Thus, our results suggest that BCDIN3D activity on tRNA^His^ could protect this tRNA from digestion by Dicer in cells, which is consistent with our observation that 5’Pme2 inhibits the generation of tRNA^His^ 3’ fragments by Dicer *in vitro* (Fig 4C).

To determine how miR-4454/tRNA^His^ 3’ fragments are produced in cells, we performed Drosha and Dicer knock downs experiments in MDA-MB-231 cells (S5A Fig). Our results showed that miR-4454/tRNA^His^ 3’ fragments behave differently from canonical miRNAs in that their levels were unaffected by Drosha knock down (S5A Fig). Unfortunately, we were unable to formally show that Dicer is responsible for the generation of tRNA^His^ 3’ fragments in MDA-MB-231 cells, because we were unable to functionally knock-down Dicer in these cells (S5A Fig). However, while the tRNA^His^ 3’ fragment is not incorporated into Ago2 (S5B Fig), analysis of tRNA^His^ 3’ fragments in the relational database of Transfer RNA related Fragments tRFdb [25] showed that the tRF with ID: 3013b, which corresponds to the tRNA^His^ 3’ fragment described here, is highly enriched in Ago3 and Ago4 PARCLIP (S5C Fig). These findings suggest that tRNA^His^ 3’ fragments may have regulatory function(s) in cells. Our result showing increased levels of tRNA^His^ 3’ fragments in MDA-MB-231 cells depleted for BCDIN3D is reproduced in Hap1 cells that have a BCDIN3D knock-out and complete loss of tRNA^His^ methylation (S6 Fig). Thus, BCDIN3D regulates the levels of tRNA^His^ 3’ fragments in at least two different cell lines. In the future, it will be of upmost importance to investigate the biological function of tRNA^His^ 3’ fragments, including in contexts where BCDIN3D function may be of clinical importance, such as cancer and metabolic disease [5, 26, 27].

## Discussion

Based on our results, we hypothesize that BCDIN3D forms an RNP with mature tRNA^His^ that is phospho-methylated (Figs 1 and 2). This could result from relatively slow product release as recently shown for the MePCE phosphomethyltransferase [28]. Stable interaction with tRNA^His^ may regulate BCDIN3D activity towards its other targets, including precursor miRNAs [5] or other yet to be uncovered phospho-methylated RNAs, by affecting BCDIN3D structure and/or RNA target selection. In this context, it will be of particular interest to investigate which cellular conditions disrupt BCDIN3D interaction with tRNA^His^, and how in turn those conditions affect BCDIN3D methyltransferase activity towards its other RNA targets. The gel shift assay for probing tRNA^His^ phospho-methylation status developed here (Fig 2) provides a fast tool for testing tRNA^His^ methylation in cells (S6 Fig), and iterations of this assay could be used for other BCDIN3D targets as well.

Our results also suggest that one of the molecular consequences of BCDIN3D-mediated methylation of tRNA^His^ is to inhibit its cleavage by Dicer (Fig 4A–4E and S6 Fig). In this context, it is intriguing that BCDIN3D downregulation or knock-out does not decrease the steady-state levels of full-length tRNA^His^ in cells (Fig 4F, S6A Fig and [16]). This may be due to compensation by higher levels of transcription at tRNA^His^ loci in shBCDIN3D cells.

Our discovery that BCDIN3D partially protects tRNA^His^ from Dicer cleavage may have evolutionary relevance. We discovered BCDIN3D in our screen for previously uncharacterized methyltransferases with human homologs conserved in fission yeast but not budding yeast [5]. Indeed, BCDIN3D and MePCE have a single homolog in fission yeast [5]. Among the known RNAs targeted by BCDIN3D and MePCE (pre-miRNA, tRNA^His^ and 7SK), only tRNA^His^ exists in fission yeast. Interestingly, Dicer is also conserved in fission yeast but not budding yeast. Dcr1, the fission yeast Dicer homolog, does not process microRNAs, but has a role in generating small interfering RNAs [29]. Thus, the function of tRNA^His^ protection from Dicer processing may be the most ancient function of the BIN3 family of 5’ phospho-methyltransferases.

BCDIN3D depletion significantly increases the levels of tRNA^His^ 3’ fragments in cells (Fig 4E and S6B Fig). It was recently shown that specific tRNA 3’ fragments limit the reverse transcription of LTR-retrotransposons in mammalian cells [10]. In this context, it is plausible that the high levels of BCDIN3D observed in aggressive breast cancers [5, 27] could decrease the levels of tRNA^His^ 3’ fragments to promote the mobility of these transposable elements and to enhance genomic evolution, which is a driving force promoting metastasis and drug resistance of cancer cells [30].

A recent paper by Hasler *et al*. showed that in La knockdown that shifts the pre-tRNA-Ile2-TTA-2-3 towards a hairpin structure, the pre-tRNA is cleaved by Dicer to generate a small RNA that acts as a microRNA (miR-1983) [31]. Thus, analogously to the mode of action of La, BCDIN3D-mediated phospho-methylation may stabilize a tRNA^His^ three-dimensional structure that counteracts Dicer processing.

Finally, hsa-miR-4454 has been detected in a number of studies aiming to identify cancer and disease biomarkers including bladder cancer [7], inflammatory bowel disease [9], and osteoarthritis [8]. Our results indicate that miR-4454 actually corresponds to a tRNA^His^ 3’ fragment. Thus it will be of high interest to re-evaluate these translational studies in the light of hsa-miR-4454 being a tRNA 3’ fragment regulated by BCDIN3D and to explore its physiological functions.

## Methods

### Cell lines

HeLa-S3-FlpIn Parental and BCDIN3Df were previously described [5]. These cells were grown in spinner flasks at 75 rpm in RPMI containing 10% fetal bovine serum (FBS), 100 U/ml penicillin, 100 μg/ml streptomycin and 2 mM L-glutamine (RPMI+10%FBS+PSQ) and supplemented with 200 μg/ml of Zeocin (parental) or 400 μg/ml hygromycin (BCDIN3Df). MDA-MB-231shBCDIN3D were generated by transfection of MDA-MB-231 cells with pRS-shBCDIN3D TR317908C/TI368844 plasmid from Origene, while the matched MDA-MB-231shNC were generated by transfection with pRS-Scrambled TR30012. These cells were grown in DMEM+10%FBS+PSQ+1 μg/ml of puromycin. The Hap1 Parental and BCDIN3D KO cells were produced by Horizon and were grown in IMDM+10%FBS+PSQ.

### Purification of BCDIN3D-interacting proteins and RNAs

2×10^7^ Hela-S3-Flp-In and Hela-S3-Flp-In-BCDIN3Df cells grown to a density of 4–6×10^5^ cells per ml were used per Co-IP. The cells were washed twice with 25 ml of cold PBS, extracted with 0.6 ml of cold co-IP buffer (20 mM HEPES pH7.5, 150 mM NaCl, 20% glycerol, 0.1% NP40, 1 mM EDTA, 0.1 mM PMSF supplemented with EDTA-free Complete Protease Inhibitor cocktail from Roche) for 1 h at 4°C and cleared by centrifugation for 10 min at 15,000 × g at 4°C. The supernatant was incubated for 4 h with 40 μl of pre-washed anti FLAG M2 conjugated beads (Sigma) at 4°C. The beads were washed 3 times with 0.6 ml of co-IP buffer, once with 0.6 ml of TBS, and eluted with 100 μl of TBS containing 150 ng/μl of 3xFLAG peptide for 30 min at 4°C. Half of the FLAG eluates were used for protein analysis and quantification, and half for RNA purification and analysis. RNA was purified using the Qiagen RNeasy MinElute Cleanup Kit with a modified protocol that allows recovery of RNAs of all sizes. 50 μl of FLAG eluates was mixed with 50 μl of water, 350 μl of RLT buffer and 675 μl of 100% molecular grade Ethanol. The mixture was passed through the Qiagen RNeasy MinElute column. The column was successively washed with 500 μl of RPE buffer and 750 μl of 80% ethanol, dried by centrifugation and the RNA was eluted twice with 17 μl of water. Proteins were migrated on a NuPAGE 4–12% Bis-Tris gel and stained with a Colloidal Blue Staining kit. Specific bands present in BCDIN3Df but not in the Control FLAG eluates were cut out using a Gene Catcher tip and sent for LC-MS/MS analysis at the Taplin Mass Spectrometry Facility at Harvard Medical School.

### Silver stain

RNA samples were separated on a denaturing 15% polyacrylamide/urea gel and stained using the FASTsilver Gel Staining Kit (#341298).

### TGIRT-seq libraries

TGIRT-seq libraries were prepared with a modification of the Total RNA-seq method [32]. Reverse transcription with TGIRT-III (InGex) was initiated from a DNA primer (5'-GTGACTGGAGTTCAGACGTGTGCTCTTCCGATCTTN-3') encoding the reverse complement of the Illumina Read2 sequencing primer binding site (R2R) annealed to a complementary RNA oligonucleotide (R2) such that there is a single nucleotide 3’ DNA overhang composed of an equimolar mixture of A, G, C and T. The RNA oligonucleotide is blocked at its 3’ end with C3Sp (IDT) to inhibit template switching to itself. Reactions contained purified RNAs, reaction medium (20 mM Tris-HCl, pH7.5, 450 mM NaCl, 5 mM MgCl_2_), 5 mM DTT, 100 nM starting annealed molecule and 1 μM TGIRT-III. Reactions were pre-incubated at room temperature for 30 min and cDNA synthesis was initiated by addition of 1 mM dNTPs (an equimolar mix of dATP, dGTP, dCTP and dTTP). Reactions were incubated at 60°C for 15 min and were terminated by adding 5 N NaOH to a final concentration of 0.25 N and incubated at 95°C for 3 min to degrade RNAs and denature protein. The reactions were then cooled to room temperature and neutralized with 5 N HCl. cDNAs were purified by using a Qiagen MinElute Reaction Cleanup Kit and then ligated at their 3’ ends to a DNA oligonucleotide encoding the reverse complement of the Illumina Read1 primer binding site (R1R) using Thermostable 5’ AppDNA/RNA Ligase (New England Biolabs). Ligated cDNAs were re-purified with MinElute Reaction Cleanup Kit and amplified by PCR for 12 cycles using Phusion DNA polymerase (Thermo Fisher Scientific) with overlapping multiplex and barcode primers that add sequences necessary for Illumina sequencing. PCR reactions were cleaned up with AMPure XP beads (Beckman Coulter) to remove adapter dimers. Libraries were sequenced on a NextSeq 500 instrument (75-nt, paired-end reads) at the Next Generation Sequencing Facility at MD Anderson Science Park.

### MicroRNA-Seq libraries

10 μg of total RNA was separated on a denaturing 15% polyacrylamide/urea gel and small RNAs < 34 nt were extracted from the gel. The rest of the library preparation was performed as in Ingolia *et al*. [11] with minor modifications.

### Bioinformatic analysis

#### Sequence pre-processing

For all samples, reads were adapter trimmed using Cutadapt v1.9.1 (with Python v2.7.3) from both the 3’ end and 5’ end, and with otherwise default parameters. Read pairs were merged using Flash v1.2.11 with the following parameters: -m 5 -M 75 -O -t 14 and only merged fragments were used for alignment.

#### Small RNA alignment

We assembled a custom human small RNA reference from several published databases, including nuclear tRNAs from tRNAdb, mitochondrial tRNAs from mitotRNAdb, piwiRNAs from piRNAdb, microRNAs from miRBase, and ribosomal RNA from annotations in the UCSC Genome Table Browser. We padded both ends of each sequence with 10 N’s (to capture non-templated end modifications), concatenated the resulting FASTA files, and built a reference genome using STAR v2.5.2b with default parameters, except for the following:—genomeSAindexNbases 7—genomeChrBinNbits 10 (to accommodate the high count and short length of human sRNA sequences). We mapped all processed libraries against this reference using STAR v2.5.2b with default parameters, except for the following:—outSAMprimaryFlag AllBestScore—seedSearchStartLmax 15—outFilterScoreMinOverLread 0.25—seedSearchLmax 15. Briefly, these parameter changes increase mapping sensitivity (due to the very short nature of each “contig”), while allowing multiple top-scoring alignments to different small RNAs to be flagged as primary (due to the repetitive nature of some small RNA sequences). Finally, we filtered all alignments marked as secondary and separated remaining alignments into reads that were greater than, or less than or equal to, 50 bp using Samtools v1.2 and custom scripts.

#### Sequence post-processing and quantitation

After separating alignments based on fragment length, we used the Sam4WebLogo package from the Jvarkit library (https://github.com/lindenb/jvarkit/wiki/SAM4WebLogo) with the “—clipped” option to generate FASTA files of uniform length for each feature of interest (miRNAs and tRNAs). We then used WebLogo v3 to generate sequence logos for these features, using the FASTA files as input, with the following parameters: -F pdf—stacks-per-line 120—color-scheme classic. We also used custom tools to extract and collapse specific sequences from those features. All downstream plotting not performed by WebLogo was performed using custom scripts in R. To quantitate each small RNA sequence, we used both Samtools v1.2 idxstats (to count reads per feature), and simple Bash scripts (to count reads per unique sequence). IGV plots were generated with the IGV_2.4.10 software using our custom small RNA reference and default parameters. For TGIRT-seq of *in vitro* Dicer assays, adapter and primer sequences were trimmed from the reads by using Cutadapt v1.16 with the following parameters: -m 15-O 5—nextseq-trim = 20—trim-n -q 20. Reads were then mapped by using Bowtie2 v2.3.4.1 with local alignment (-k 1—very-sensitive-local—norc—no-mixed) to the synthetic mature tRNA^His^-_GUG_ sequence, 5’- GGCCGTGATCGTATAGTGGTTAGTACTCTGCGTTGTGGCCGCAGCAACCTCGGTTCGTAT CCGAGTCACGGCACCA-3’. For coverage plot and read length distribution plot, reference sequence coverages and read pair length were calculated using BEDTools v2.27.1.

### RNA methyltransferase assays

RNA methyltransferase assays with BCDIN3D were performed in a total volume of 100 μl in 50 mM Tris-HCl, pH8; 150 mM NaCl; 1 mM EDTA; 5 mM DTT; 20% glycerol; 4 μl of ^3^H-SAM (PerkinElmer NET155250UC); 1X EDTA-free Complete Protease Inhibitor cocktail from Roche, 80 U of RNaseOUT from Invitrogen with 1 μg of recombinant BCDIN3D and 1 μl of 100 μM synthetic RNA for 2h at 37°C.

### tRNA^His^ guanylation assays

tRNA^His^ guanylation assays with THG1L were performed in a total volume of 40 μl in 25 mM HEPES, pH7.4; 125 mM NaCl; 10 mM MgCl_2_, 3 mM DTT; 3 mM ATP, 0.8 μM tRNA^His^, 0.4 μM ^32^Pα-GTP and 0.4 μM recombinant THG1L for 1h30 at 25°C.

### Treatments with Antarctic Phosphatase and Polynucleotide Kinase

Treatments with Antarctic Phosphatase or T4 Polynucleotide Kinase (New England Biolabs) were performed on 100 pmol of synthetic RNAs or on 100 ng of RNA purified from FLAG eluates. The reactions were performed in a total volume of 20 μl of 1X Antarctic Phosphatase Reaction Buffer (50 mM Bis-Tris-Propane-HCl, 1 mM MgCl2, 0.1 mM ZnCl2, pH 6 @ 25°C) with 5 units of Antarctic Phosphatase or 20 units of T4 Polynucleotide Kinase or mock for 30 min at 37°C.

### RNA/Protein extraction

Total RNA and protein extraction was typically performed on ~ 5x10^5^ cells using the RNA/Protein Plus purification kit from Norgen (Product # 48000). Cells grown on 3 cm diameter dishes were washed with 2 ml of PBS and lysed with 300 μl of Lysis Buffer supplemented with 10 μl of β-mercaptoethanol per ml for 5 min on a rocking table. RNA extraction was performed according to the manufacturer’s instructions. The whole flow-through after the RNA binding step was used for protein purification. The RNA and protein concentrations were measured with a Denovix device.

For the purification of charged tRNAs, 10^6 cells were resuspended in 300 μl of cold Lysis Buffer solution (0.3 M Sodium Acetate pH 5.2, 10 mM EDTA) and extracted with 300 μl of cold acetate-saturated Phenol/Chloroform (pH 4.5). 250 μl of the aqueous upper layer was mixed with 675 μl of cold ethanol and centrifuged for 1 h at 18,600 rcf at 4°C. The RNA pellet was resuspended in 50 μl of RNA Resuspension Solution (10 mM Sodium Acetate pH 5.2, 1 mM EDTA). To deacylate a portion of the tRNA, 1 μg of RNA was treated with 0.1M Tris-HCl, pH 9.5 for 30 min at 37°C.

### RNA analysis

Taqman RTqPCR was performed with the Taqman MicroRNA Reverse Transcription Kit from Applied Biosystems. 500 ng of total RNA was used for reverse transcription of mRNAs with the SuperScript III First-Strand Synthesis System from invitrogen. Real-time PCR analysis was performed on a StepOne Plus system. The Northern Blots were performed as previously described [33]. Small DNA ladders were used as markers in our denaturing 15% polyacrylamide/urea gels, either the 10 bp DNA Ladder (#10821–015) from Invitrogen, or the ss20 ssDNA Ladder from Simplex sciences. Please note that the ssDNA ladder bands are offset by ~10–20 nt compared to RNA.

### Chromatin immunoprecipitation

ChIP-Seq experiments and bioinformatic analysis was performed as previously described [34].

### Dicer assays

20 pmol of synthetic RNA was incubated with the indicated volumes of human recombinant Dicer (Invitrogen # K3600-01 or Origene TP319214) in a total volume of 15 μl in 100 mM KCl, 10 mM Tris-HCl, pH8, 0.1 mM EDTA, 1 mM MgCl_2,_ 0.5 mM dTT supplemented with 0.5 U/μl RNaseOUT for 2h at 37°C. The samples were mixed with 15 μl of Gel Loading Buffer II (Ambion), heated for 15 min at 70°C and separated on a denaturing 15% polyacrylamide/urea gel. The gels were stained for 5 min with 1x SYBR Gold and the signal was detected and analyzed with the G:Box gel doc system from Syngene.

### X-RIP

X-RIP was performed as previously described [5].

## Supporting information

S1 Table TGIRT-seq results from RNA purified out of FLAG eluates of HeLa-S3-FlpIn Parental and BCDIN3Df as in Fig 1A–1C (reads > 50 nt). 2 biological repeats are shown.
(XLSX)

S2 Table Mass spectrometry results for BCDIN3Df TRMT5 protein interactor. (DOCX)

S3 Table TGIRT-seq results from RNA purified out of FLAG eluates of HeLa-S3-FlpIn Parental and BCDIN3Df as in Fig 1A–1C (reads < 50 nt). 2 biological repeats are shown.
(XLSX)

S4 Table List of oligonucleotides. (DOCX)

S5 Table List of synthetic RNAs. (DOCX)

S6 Table List of antibodies. (DOCX)

S7 Table Numerical data. (XLSX)

S1 Fig Schematic of the bioinformatic pipeline used to analyze the TGIRT-seq libraries from the RNA purified out of FLAG eluates of HeLa-S3-FlpIn Control and BCDIN3Df. (PNG)

S2 Fig A. Number of reads < 50nt mapping to tRNA^His^ in two biological replicates of Control and BCDIN3Df FLAG eluates. B. The sequences of BCDIN3D interacting RNAs <50nt mapping to tdbD00005717_His_GTG (tRNA^His^) are represented in a WebLogo format. These reads could be due to an m1G reverse transcriptase roadblock at G37. (C) 5 μl of Hela-S3-Flp-In Control and BCDIN3Df inputs and FLAG eluates were analyzed by western blot with a TRMT5 antibody.
(PNG)

S3 Fig Validation of the TGIRT-seq results from RNA purified out of FLAG eluates of HeLa-S3-FlpIn Parental and BCDIN3Df with probes for U6 (background RNA, present in both samples) and hsa-miR-4454 (enriched in BCDIN3Df interacting RNAs). Please note that the y-axis is in log_10_ scale.
(PNG)

S4 Fig A. hsa-miR-4454 stem loop as annotated in miRBase, with the mature miR-4454 sequence highlighted in red. B. UCSC genome browser traces of the H3K4me3 ChIP-Seq in HeLa-S3-Flp-In cells at the annotated hsa-miR-4454 locus, and as a positive control, at the TSS (Transcription Start Site) of the active hsa-let-7a-1/7d/7f-1 primary miRNA. H3K4me3 is a histone modification that marks the TSS of transcriptionally active genes. C. Screenshot of miR-4454 stemloop in UCSC genome browser (hg38 genome assembly) showing its position with respect to HERVH-int. D. Shown are hsa-miR-4454 reads from miRBase and from BCDIN3Df FLAG eluates (repeats #1 and 2). The IVG plot shows the base identity of all hsa-miR-4454 reads from BCDIN3Df FLAG eluates at position G4 of the hsa-miR-4454 stem loop, which aligns to the A57 residue of tRNA^His^. The spectrum of incorporated nucleotides, dominated by T and G, is characteristic of TGIRT-III misincorporation at m1A [23].
(PNG)

S5 Fig A. MDA-MB-231 cells were reverse transfected with 50 nM siNC, siDrosha and siDicer at three consecutive timepoints (0h, 24h, 72h) and total RNA from these cells was purified at the 96h timepoint. The graph on the left shows the RTqPCR analysis of the Drosha and Dicer mRNA normalized to ALAS1, B2M and siNC, and the graph on the right shows the Taqman RTqPCR analysis of select miRNAs and U6 normalized to siNC. Shown are mean ± SD (n = 2 biological replicates). Note: After these 3 rounds of siRNA transfection, 90% reduction of Dicer levels are not sufficient to downregulate miR-21, which is the major miRNA in MDA-MB-231 cells. Additionally, other miRNAs are only mildly affected by Dicer knock-down. In contrast, Drosha knock-down has the expected effect on these bona fide miRNAs. This suggests that very low levels of Dicer are sufficient for its function. Additionally, the fact that Drosha knock-down does not affect miR-4454 levels further supports the idea that miR-4454 is not generated from a primary miRNA, but from mature tRNA^His^ cleavage. B. hsa-miR-4454/3’ tRNA^His^ fragment do not associate with Ago2. MDA-MB-231shNC and shBCDIN3D cells were subjected to crosslinked RNA-immunoprecipitation (X-RIP) with anti-GFP and anti-Ago2 antibodies. RNA from the immunoprecipitates was purified and the levels of miR-21 and 3’ tRNA^His^ fragment/hsa-miR-4454 were analyzed by Taqman RT-qPCR. The data are normalized to the U6 RNA and to the shNC-input sample. Shown are mean ± SD (n = 3 technical replicates) of a representative example (n = 2 biological replicates). C. Analysis of tRNA^His^ 3’ fragment (tRF with ID: 3013b) enrichment in Ago1-4-FLAG PARCLIP data in the relational database of Transfer RNA related Fragments (tRFdb).
(PNG)

S6 Fig A. Total RNA purified from Hap1-WT and BCDIN3D-KO cells were treated with mock or Antarctic Phosphatase (AP), separated on a denaturing 15% polyacrylamide/urea gel, and probed with the tRNA^His^ northern blot probe #2. The treatment with AP shifted the migration of tRNA^His^ in BCDIN3D-KO cells, but not in WT cells, showing that tRNA^His^ is fully phosphomethylated in Hap1 cells and fully unmethylated when BCDIN3D is knocked out. Note that the ladder is the ss20 ssDNA Ladder and its migration is offset by 10–20 nt compared to RNA. B. Taqman RTqPCR analysis of 3’ tRNA^His^ fragment/hsa-miR-4454 in Hap1-WT and BCDIN3D-KO cells. Shown are mean ± SEM (n = 2 biological replicates).
(PNG)
