# Supplementary material for: BCDIN3D regulates tRNAHis 3’ fragment processing
Source: PLoS Genet. 2019 Jul 22;15(7):e1008273. doi: 10.1371/journal.pgen.1008273 (PMC6675128; doi:10.1371/journal.pgen.1008273)
Supplement: S2 Table — (DOCX) [file pgen.1008273.s002.docx]

**S2 Table.** Mass spectrometry results for BCDIN3Df TRMT5 protein interactor.

| **Unique Peptides** | **Total Peptides** | **Reference** | **Average** | **Gene Symbol** | **MWT (kDa)** |
| --- | --- | --- | --- | --- | --- |
| 11 | 12 | Q32P41_TRM5_HUMAN | 2.9172 | TRMT5 | 58.21 |
