# Supplementary material for: BCDIN3D regulates tRNAHis 3’ fragment processing
Source: PLoS Genet. 2019 Jul 22;15(7):e1008273. doi: 10.1371/journal.pgen.1008273 (PMC6675128; doi:10.1371/journal.pgen.1008273)
Supplement: S4 Table — (DOCX) [file pgen.1008273.s004.docx]

**S4 Table.** List of oligonucleotides.

| **Primers** | **Code** | **Sequence** |
| --- | --- | --- |
| **tRNA^His^ probe #1** | BX00051 | TCGGATACGAACCGAGGTTGC |
| **tRNA^His^ probe #2** | BX00050 | TGGTGCCGTGACTCGGATAC |
| **U6_probe** | TK10027 | GCAGGGGCCATGCTAATCTTCTCTGTATCG |
| **Custom hsa-miR-4454 Taqman^®^ microRNA assay** | Custom target sequence miR-4454: CGGAUCCGAGUCACGGCACCA, Catalog #: 4427975 | |
| **U6 Taqman® microRNA assay** | Assay ID: 000400, Catalog #: 4427975 | |
| **hsa-miR-21 Taqman® microRNA assay** | Assay ID: 000397, Catalog #: 4427975 | |
| **hsa-miR-23b Taqman® microRNA assay** | Assay ID: 001973, Catalog #: 4427975 | |
| **hsa-let-7f Taqman® microRNA assay** | Assay ID: 000382, Catalog #: 4427975 | |
| **BCDIN3D_FWD** | BX00070 | GCCCCGTTCGGAAATTTTC |
| **BCDIN3D_REV** | BX00071 | ACACTCAGATCCCCGGAGTTAC |
| **ALAS1_ FWD** | BX00060 | CCTTGGCCTTAGCAGTTTTG |
| **ALAS1_ REV** | BX00061 | CCAAGATGATGGAAGTTGGG |
| **B2M_FWD** | BX00062 | AATGTCGGATGGATGAAACC |
| **B2M_REV** | BX00063 | TCTCTCTTTCTGGCCTGGAG |
| **Drosha_ FWD** | BX00530 | AAGGAAGCTGGCAAACAAGA |
| **Drosha_ REV** | BX00531 | AAAACGAACCACCAAGTTGC |
| **Dicer_ FWD** | BX00532 | ATCGGTTGTTCCTGAACCTG |
| **DICER_ REV** | BX00533 | TCGGTATTTTGCCTGTCCTC |
