# Supplementary material for: BCDIN3D regulates tRNAHis 3’ fragment processing
Source: PLoS Genet. 2019 Jul 22;15(7):e1008273. doi: 10.1371/journal.pgen.1008273 (PMC6675128; doi:10.1371/journal.pgen.1008273)
Supplement: S5 Table — (DOCX) [file pgen.1008273.s005.docx]

**S5 Table.** List of synthetic RNAs.

| **RNA** | **Sequence** |
| --- | --- |
| **pre-miR-145-5’OH** | 5’OH -GUCCAGUUUUCCCAGGAAUCCCUUAGAUGCUAAGAUGGGGAUUCCUGGAAAUACUGUUCU |
| **pre-miR-145-5’P** | 5’PO_4_ -GUCCAGUUUUCCCAGGAAUCCCUUAGAUGCUAAGAUGGGGAUUCCUGGAAAUACUGUUCU |
| **pre-miR-145-5’Pme1** | 5’Pme1 -GUCCAGUUUUCCCAGGAAUCCCUUAGAUGCUAAGAUGGGGAUUCCUGGAAAUACUGUUCU |
| **pre-miR-145-5’Pme2** | 5’Pme2 -GUCCAGUUUUCCCAGGAAUCCCUUAGAUGCUAAGAUGGGGAUUCCUGGAAAUACUGUUCU |
| **tRNA^His^noG_-1_-5’P** | 5'P-  GCCGUGAUCGUAUAGUGGUUAGUACUCUGCGUUGUGGCCGCAGCAACCUCGGUUCGUAUCCGAGUCACGGCACCA -3' |
| **tRNA^His^-5’OH** | 5'OH-  GGCCGUGAUCGUAUAGUGGUUAGUACUCUGCGUUGUGGCCGCAGCAACCUCGGUUCGUAUCCGAGUCACGGCACCA -3' |
| **tRNA^His^-5’P** | 5'P- GGCCGUGAUCGUAUAGUGGUUAGUACUCUGCGUUGUGGCCGCAGCAACCUCGGUUCGUAUCCGAGUCACGGCACCA -3' |
| **tRNA^His^-5’Pme2** | 5'Pme2- GGCCGUGAUCGUAUAGUGGUUAGUACUCUGCGUUGUGGCCGCAGCAACCUCGGUUCGUAUCCGAGUCACGGCACCA -3' |
| **siNC** | Dharmacon D-001810-10-20, ON-Target plus Non-targeting Pool |
| **siDrosha** | Dharmacon L-016996-00-05, ON-Target plus SMARTpool RNASEN siRNA |
| **siDicer** | Dharmacon L-003483-00-05, ON-Target plus SMARTpool human DICER siRNA |
