# Supplementary material for: BCDIN3D regulates tRNAHis 3’ fragment processing
Source: PLoS Genet. 2019 Jul 22;15(7):e1008273. doi: 10.1371/journal.pgen.1008273 (PMC6675128; doi:10.1371/journal.pgen.1008273)
Supplement: S6 Table — (DOCX) [file pgen.1008273.s006.docx]

**S6 Table.** List of antibodies.

| **Antibody** | **Provider** | **Catalog number** | **Lot**  **number** |
| --- | --- | --- | --- |
| Anti-BCDIN3D | Abcam | ab77034 | GR43088-2 |
| Anti-Tubulin α | Abcam | ab123918 | GR164056-4 |
| Anti-TRMT5 | Proteintech | 18255-1-AP |  |
| Anti-GFP | Invitrogen | A11122 | 779558 |
| Anti-Ago2 | Cell Signaling | #2897 | 2 |
