# Supplementary figures and images for: BCDIN3D regulates tRNAHis 3’ fragment processing
Source: PLoS Genet. 2019 Jul 22;15(7):e1008273. doi: 10.1371/journal.pgen.1008273 (PMC6675128; doi:10.1371/journal.pgen.1008273)

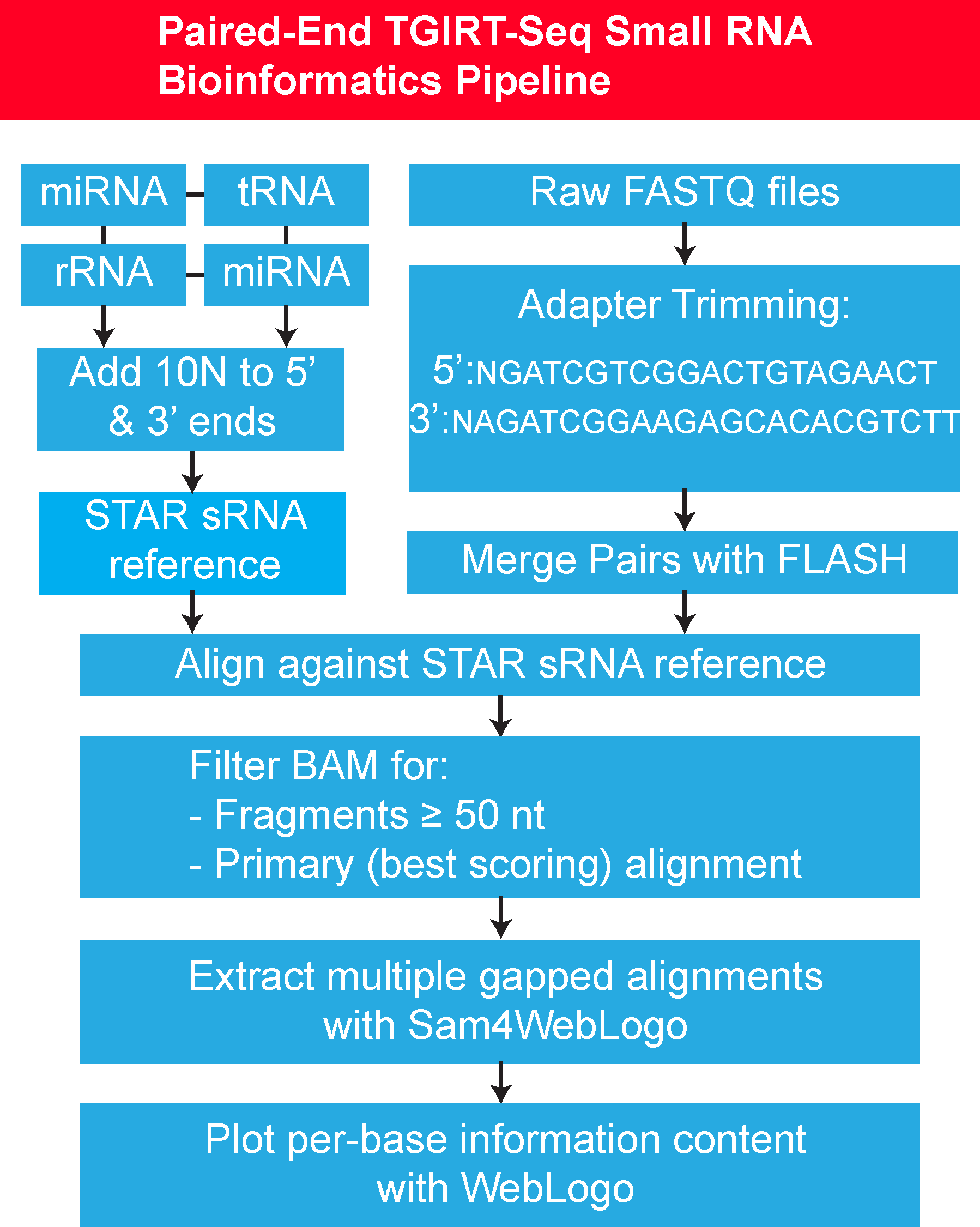

Supplement: S1 Fig — (PNG) [file pgen.1008273.s008.png]

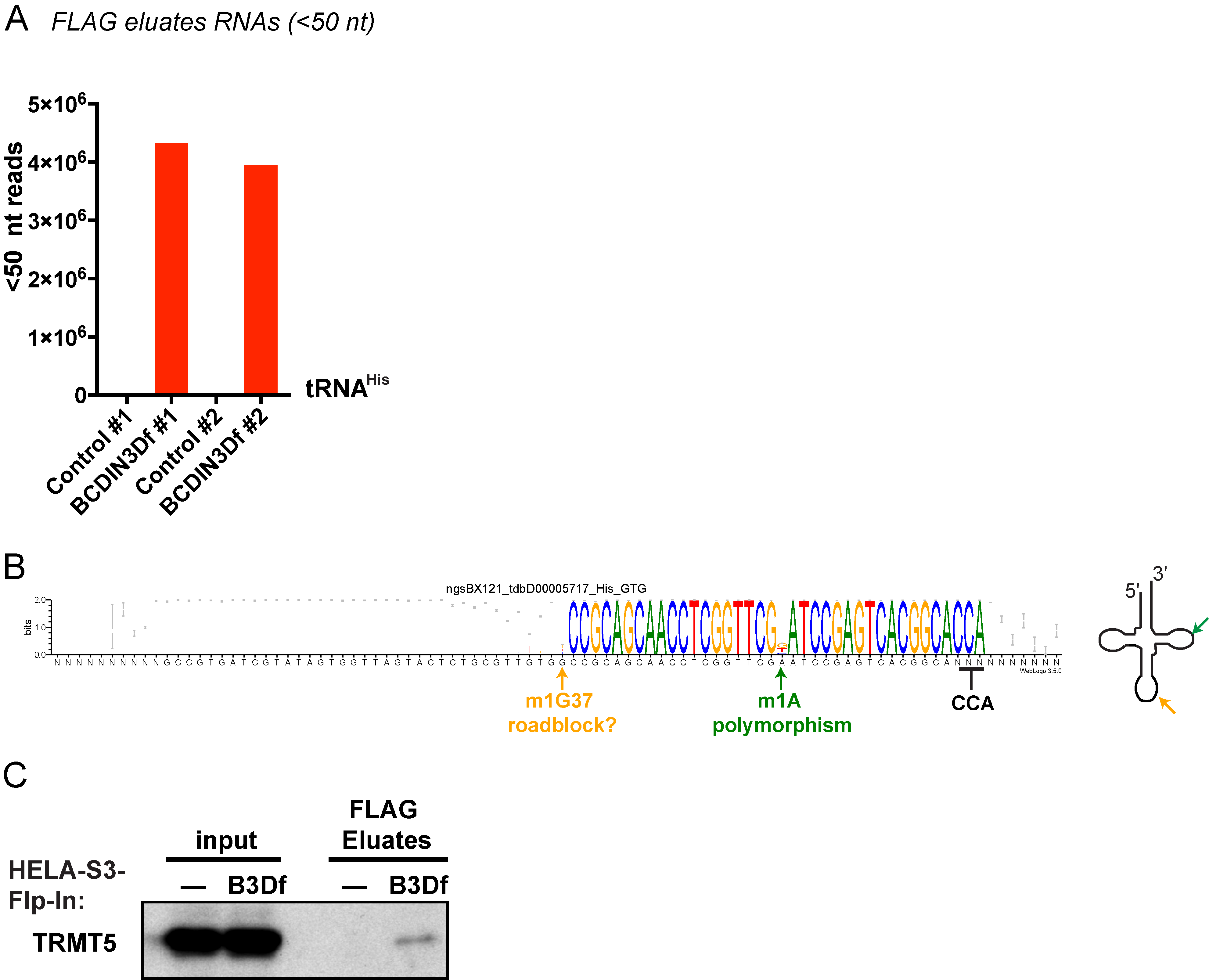

Supplement: S2 Fig — A. Number of reads < 50nt mapping to tRNAHis in two biological replicates of Control and BCDIN3Df FLAG eluates. B. The sequences of BCDIN3D interacting RNAs <50nt mapping to tdbD00005717_His_GTG (tRNAHis) are represented in a WebLogo format. These reads could be due to an m1G reverse transcriptase roadblock at G37. (C) 5 μl of Hela-S3-Flp-In Control and BCDIN3Df inputs and FLAG eluates were analyzed by western blot with a TRMT5 antibody. (PNG) [file pgen.1008273.s009.png]

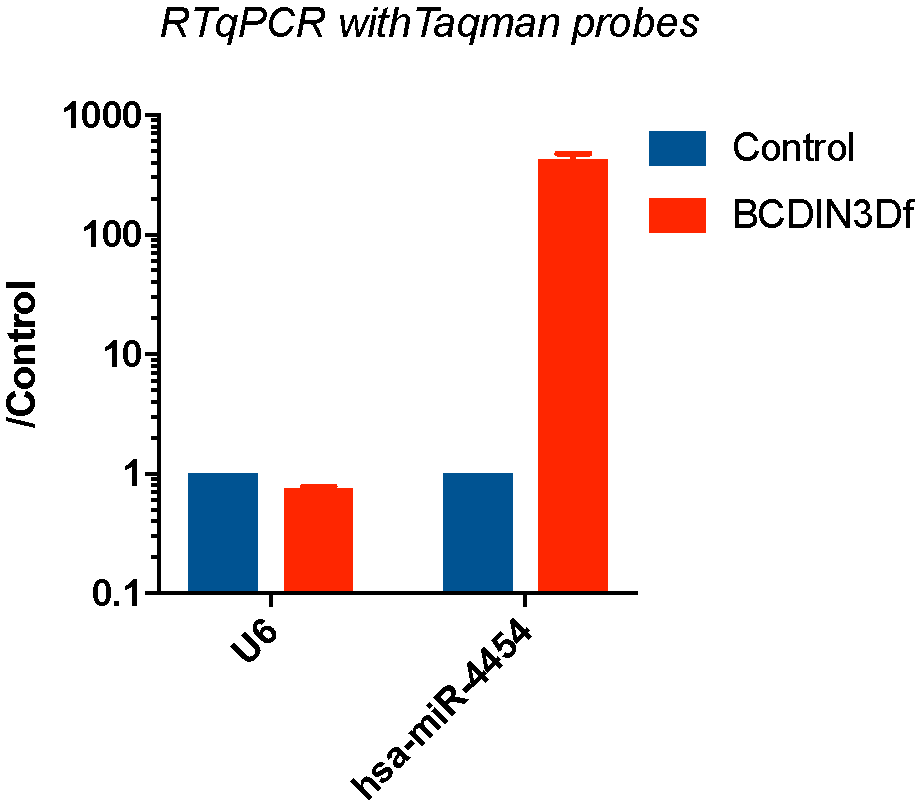

Supplement: S3 Fig — Please note that the y-axis is in log10 scale. (PNG) [file pgen.1008273.s010.png]

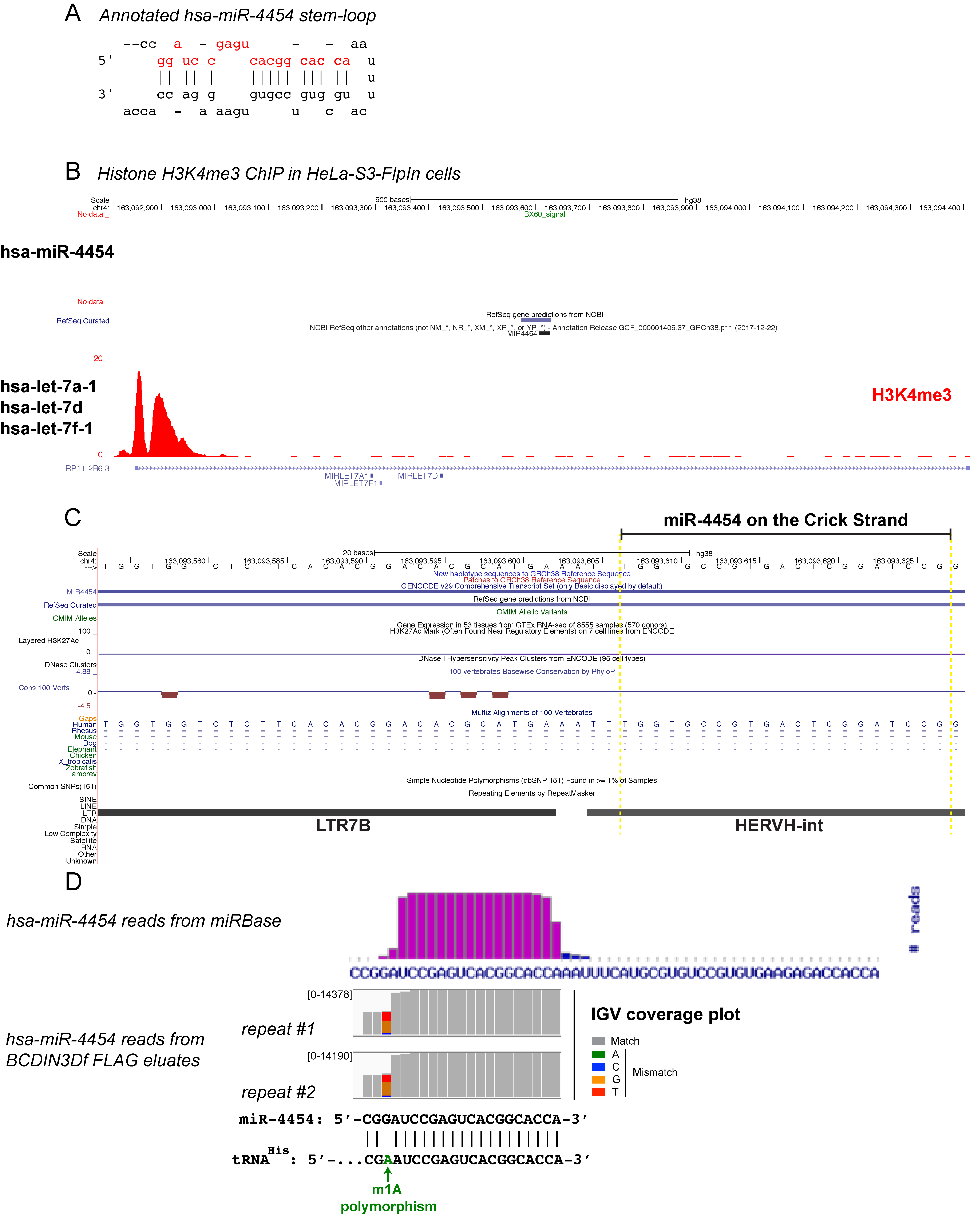

Supplement: S4 Fig — A. hsa-miR-4454 stem loop as annotated in miRBase, with the mature miR-4454 sequence highlighted in red. B. UCSC genome browser traces of the H3K4me3 ChIP-Seq in HeLa-S3-Flp-In cells at the annotated hsa-miR-4454 locus, and as a positive control, at the TSS (Transcription Start Site) of the active hsa-let-7a-1/7d/7f-1 primary miRNA. H3K4me3 is a histone modification that marks the TSS of transcriptionally active genes. C. Screenshot of miR-4454 stemloop in UCSC genome browser (hg38 genome assembly) showing its position with respect to HERVH-int. D. Shown are hsa-miR-4454 reads from miRBase and from BCDIN3Df FLAG eluates (repeats #1 and 2). The IVG plot shows the base identity of all hsa-miR-4454 reads from BCDIN3Df FLAG eluates at position G4 of the hsa-miR-4454 stem loop, which aligns to the A57 residue of tRNAHis. The spectrum of incorporated nucleotides, dominated by T and G, is characteristic of TGIRT-III misincorporation at m1A [23]. (PNG) [file pgen.1008273.s011.png]

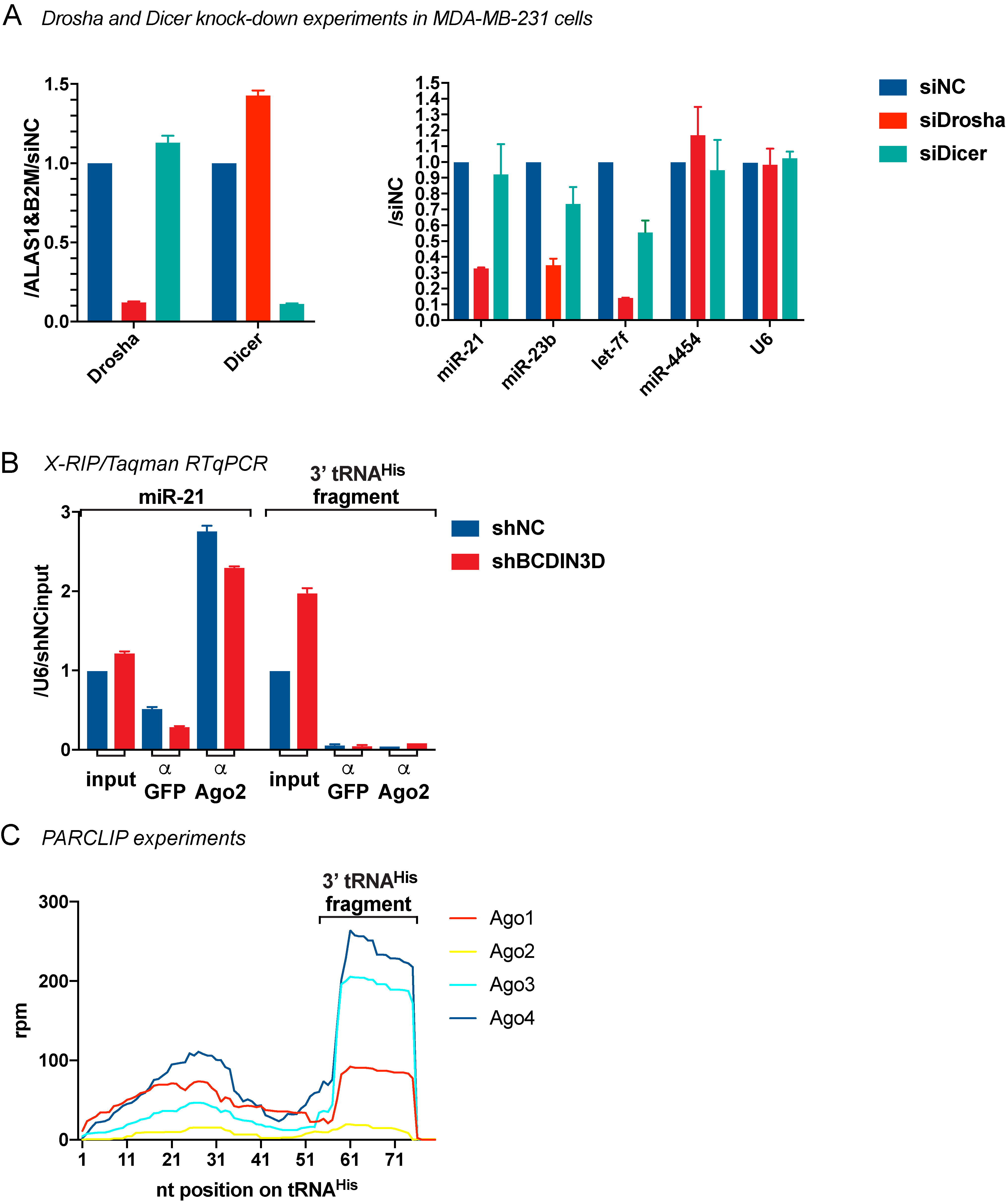

Supplement: S5 Fig — A. MDA-MB-231 cells were reverse transfected with 50 nM siNC, siDrosha and siDicer at three consecutive timepoints (0h, 24h, 72h) and total RNA from these cells was purified at the 96h timepoint. The graph on the left shows the RTqPCR analysis of the Drosha and Dicer mRNA normalized to ALAS1, B2M and siNC, and the graph on the right shows the Taqman RTqPCR analysis of select miRNAs and U6 normalized to siNC. Shown are mean ± SD (n = 2 biological replicates). Note: After these 3 rounds of siRNA transfection, 90% reduction of Dicer levels are not sufficient to downregulate miR-21, which is the major miRNA in MDA-MB-231 cells. Additionally, other miRNAs are only mildly affected by Dicer knock-down. In contrast, Drosha knock-down has the expected effect on these bona fide miRNAs. This suggests that very low levels of Dicer are sufficient for its function. Additionally, the fact that Drosha knock-down does not affect miR-4454 levels further supports the idea that miR-4454 is not generated from a primary miRNA, but from mature tRNAHis cleavage. B. hsa-miR-4454/3’ tRNAHis fragment do not associate with Ago2. MDA-MB-231shNC and shBCDIN3D cells were subjected to crosslinked RNA-immunoprecipitation (X-RIP) with anti-GFP and anti-Ago2 antibodies. RNA from the immunoprecipitates was purified and the levels of miR-21 and 3’ tRNAHis fragment/hsa-miR-4454 were analyzed by Taqman RT-qPCR. The data are normalized to the U6 RNA and to the shNC-input sample. Shown are mean ± SD (n = 3 technical replicates) of a representative example (n = 2 biological replicates). C. Analysis of tRNAHis 3’ fragment (tRF with ID: 3013b) enrichment in Ago1-4-FLAG PARCLIP data in the relational database of Transfer RNA related Fragments (tRFdb). (PNG) [file pgen.1008273.s012.png]

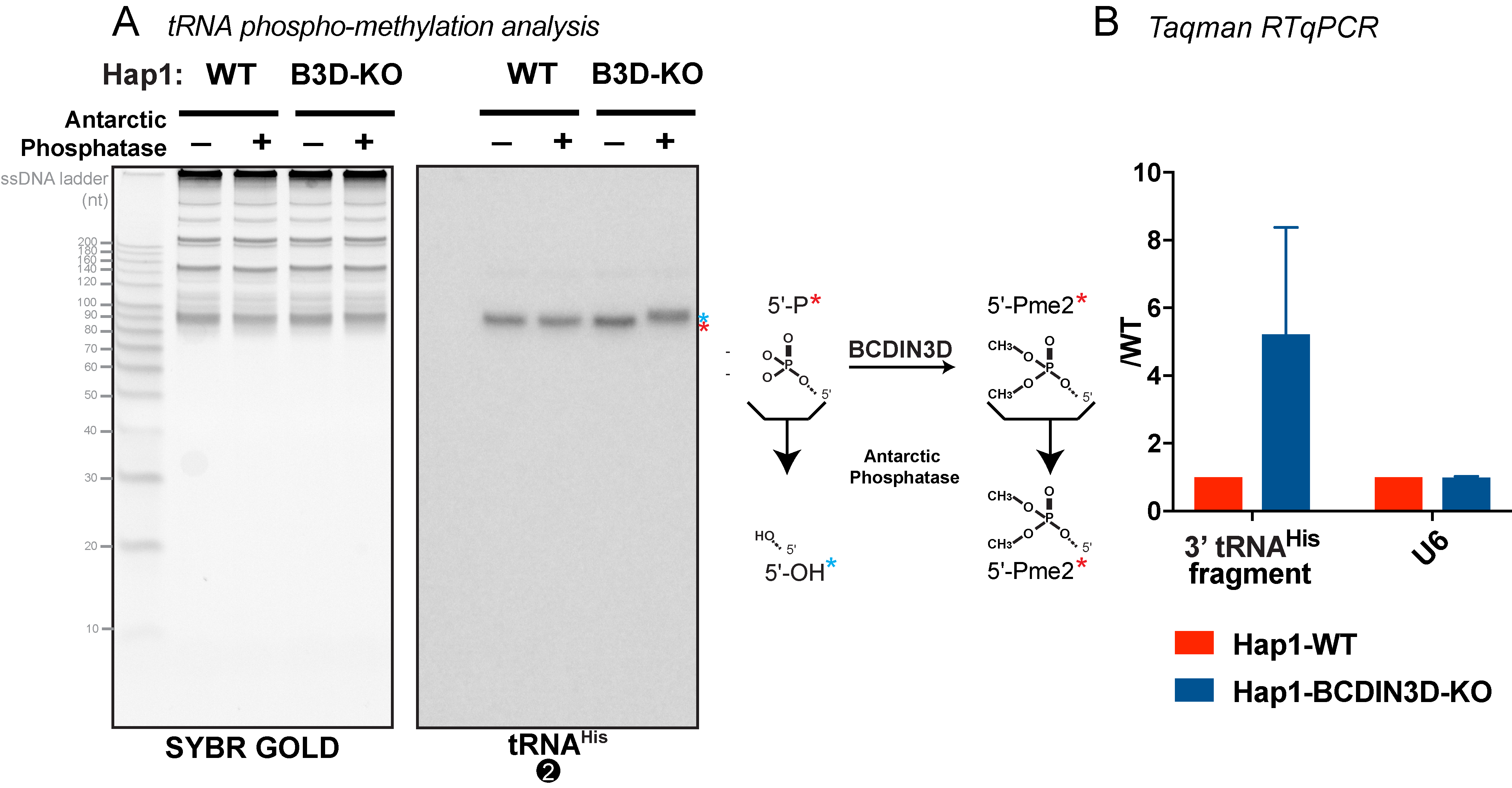

Supplement: S6 Fig — A. Total RNA purified from Hap1-WT and BCDIN3D-KO cells were treated with mock or Antarctic Phosphatase (AP), separated on a denaturing 15% polyacrylamide/urea gel, and probed with the tRNAHis northern blot probe #2. The treatment with AP shifted the migration of tRNAHis in BCDIN3D-KO cells, but not in WT cells, showing that tRNAHis is fully phosphomethylated in Hap1 cells and fully unmethylated when BCDIN3D is knocked out. Note that the ladder is the ss20 ssDNA Ladder and its migration is offset by 10–20 nt compared to RNA. B. Taqman RTqPCR analysis of 3’ tRNAHis fragment/hsa-miR-4454 in Hap1-WT and BCDIN3D-KO cells. Shown are mean ± SEM (n = 2 biological replicates). (PNG) [file pgen.1008273.s013.png]
